# Differential Binding of Tenofovir and Adefovir to Reverse Transcriptase of Hepatitis B Virus

**DOI:** 10.1371/journal.pone.0106324

**Published:** 2014-09-02

**Authors:** Formijn J. van Hemert, Ben Berkhout, Hans L. Zaaijer

**Affiliations:** 1 Laboratory of Experimental Virology, Department of Medical Microbiology, Center for Infection and Immunity Amsterdam (CINIMA), Academic Medical Center, University of Amsterdam, Amsterdam, the Netherlands; 2 Laboratory of Clinical Virology, Department of Medical Microbiology, Center for Infection and Immunity Amsterdam (CINIMA), Academic Medical Center, University of Amsterdam, Amsterdam, the Netherlands; University of Pittsburgh, United States of America

## Abstract

**Introduction:**

Resistance of the reverse transcriptase (RT) of hepatitis B virus (HBV) to the tenofovir nucleotide drug has not been observed since its introduction for treatment of hepatitis B virus (HBV) infection in 2008. In contrast, frequent viral breakthrough and resistance has been documented for adefovir. Our computational study addresses an inventory of the structural differences between these two nucleotide analogues and their binding sites and affinities to wildtype (wt) and mutant RT enzyme structures based on *in silico* modeling, in comparison with the natural nucleotide substrates.

**Results:**

Tenofovir and adefovir only differ by an extra CH_3_-moiety in tenofovir, introducing a center of chirality at the carbon atom linking the purine group with the phosphates. (R)-Tenofovir (and not (S)-tenofovir) binds significantly better to HBV-RT than adefovir. “Single hit” mutations in HBV-RT associated with adefovir resistance may affect the affinity for tenofovir, but to a level that is insufficient for tenofovir resistance. The RT-Surface protein gene overlap in the HBV genome provides an additional genetic constraint that limits the mutational freedom required to generate drug-resistance. Different pockets near the nucleotide binding motif (YMDD) in HBV-RT can bind nucleotides and nucleotide analogues with different affinities and specificities.

**Conclusion:**

The difference in binding affinity of tenofovir (more than two orders of magnitude in terms of local concentration), a 30x higher dosage of the (R)-tenofovir enantiomer as compared to conformational isomeric or rotameric adefovir, and the constrained mutational space due to gene overlap in HBV may explain the absence of resistance mutations after 6 years of tenofovir monotherapy. In addition, the computational methodology applied here may guide the development of antiviral drugs with better resistance profiles.

## Introduction

The pharmaceuticals tenofovir and adefovir are nucleotide-analogues for treatment of hepatitis B virus (HBV) infection [1], [2]. Monotherapy using adefovir frequently caused viral breakthrough due to the appearance of resistance mutations [3], [4]. In contrast, mutations conferring HBV resistance to tenofovir have not yet been described, despite 6 years of tenofovir monotherapy [5]. The molecular structure of the adefovir and tenofovir nucleotide analogues differs by an CH_3_-moiety in tenofovir instead of a hydrogen atom in adefovir at the carbon atom linking the nuclide group with the phosphates. This seemingly minor alteration introduces a chiral center at the C-atom involved. Consequently, (R)-tenofovir resembles the natural occurring beta-D-NTPs ((d)NTP  =  (deoxy)nucleosidetriphosphate) more than the (S)-tenofovir enantiomer, while adefovir may be regarded as a collection of rotamers. Most DNA polymerases, including the HBV and HIV reverse transcriptase (RT) enzymes, incorporate only natural beta-D-dNTPs into the growing DNA chain [6]. Hence, (R)-tenofovir may be better equipped structurally to inhibit DNA chain elongation at the level of dNTP recognition and binding by the viral RT enzyme than adefovir. The canonical polymerase YMDD amino acid motif in RT plays a key role in the process of nucleotide binding [7].

We challenged the RT enzyme of HBV (HBV-RT) *in silico* for its capability to bind adefovir, (R)- and (S)-tenofovir and the natural dNTPs by means of YMDD-directed docking of substrates into the 3D-model of HBV-RT [4]. We determined the energy profiles of the protein-ligand interactions (PEARLS server, [8]). We found a high affinity of HBV-RT for (R)-tenofovir, a slightly lower value for adefovir and a much lower value for (S)-tenofovir, the latter result confirming the validity of this *in silico* exercise. RT mutations conferring adefovir resistance displayed multiple effects: diminished adefovir affinity and enhancing the affinity for natural substrates. Single amino acid replacements that can decrease (R)-tenofovir binding comparable to that of dNTPs were hardly observed. The concerted action of two amino acid substitutions may obviously further decrease the affinity for (R)-tenofovir, but likely interferes with HBV fitness due to the overlap of the RT and S genes in the condensed viral genome. Nucleotide substitutions specifying resistance related amino acid replacements in the RT reading frame are attained by “difficult” transversions, arguing that “easy” transitions are not compatible with enzyme activity or drug resistance of the RT-enzyme [9]. We also show that detrimental effects in the overlapping S reading frame are effectively avoided. *In silico* docking experiments of tenofovir, adefovir and dNTPs into HBV-RT indicate that multiple sites near the YMDD binding motif interact with these drugs and the natural nucleotides.

## Results

### 1. Differential binding of adefovir and tenofovir to HBV-RT

**Figure 1:**
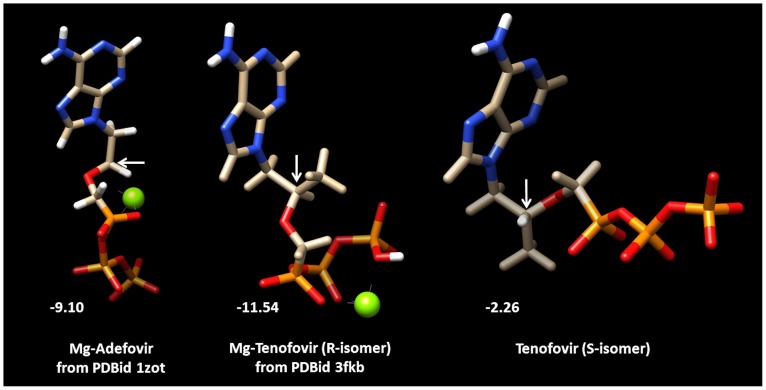
Structures of adefovir, (R)-tenofovir and (S)-tenofovir. PDB coordinates of Mg-adefovir and Mg-(R)-tenofovir were taken from the PDBids 1ZOT and 3FKB, respectively. (S)-tenofovir was created via CORINA. Mg-ions near the red phosphate moieties are in green. Arrows indicate the carbon atom linking the purine group with the phosphates (a center of asymmetry in tenofovir). Numbers in white are the total protein-ligand energy interaction values indicating the affinity to wt HBV reverse transcriptase.

PDB coordinates of Mg-adefovir and Mg-(R)-tenofovir structures were taken from biologically active protein complexes of which X-ray structures have been determined (see Materials & methods). These structures show a similar intramolecular orientation of the purine and phosphate moieties and a high *in silico* determined affinity was scored as the total ligand-receptor interaction energy (in Kcal/mol) for the HBV-RT enzyme (Figure 1). It should be noted that the measure of affinity is logarithmically related to the equilibrium constant of the enzyme-substrate reaction involved. Consequently, the difference between (R)-tenofovir (−11.54 Kcal/mol) and adefovir (−9.10 Kcal/mol) binding is pronounced when considering the local drug concentration that yields 50% inhibition. The RT inhibitory activity of (S)-tenofovir (−2.26 Kcal/mol) is close to zero. Henceforth, we will skip (S)-tenofovir in the subsequent analysis and simply refer to (R)-tenofovir as tenofovir, unless specified otherwise.

Adefovir and tenofovir are purine (ATP and GTP) analogues differing by a center of asymmetry that is present exclusively in tenofovir. We compared their *in silico* RT binding activities with those of the natural purines in 3D-models of HBV-RT, either the wild-type (wt) or mutants carrying adefovir-resistance mutations (Table 1). The N53T amino acid replacement is able to reduce the affinity of tenofovir, adefovir and dGTP (not dATP) by about 0.8 Kcal/mol, which seems sufficient for adefovir resistance, but insufficient for tenofovir resistance. The S78T substitution shows a large affinity loss for tenofovir (−11.54 to −7.48), a moderate reduction for dATP (−7.40 to −5.95) and dGTP (−9.41 to −7.01) and a relatively minor decrease for adefovir (−9.10 to −8.16). These data predict that tenofovir resistance instead of adefovir resistance is observed, provided that a single site model accurately describes the HBV-RT binding of nucleotides and competing drugs. As will be argued below, this may not be the case. The S85F substitution that is associated with lamivudine-resistance reduces the dATP, dGTP and adefovir affinities, but not that of tenofovir. Diverse mutations at the A181 residue may affect nucleotide and drug affinities of HBV-RT. The A181I substitution particularly diminishes tenofovir affinity, but this change requires a double nucleotide change in the codon involved, potentially with a large impact on viral fitness (see below). A181T provides an example of adefovir resistance, showing a reduction of affinity by more than 5 Kcal/mol. Tenofovir affinity is diminished from −11.54 to −8.28 Kcal/mol, which is probably not sufficient for successful competition with dATP (−7.33) and/or dGTP (−7.11). Interestingly, the A181V mutation does not affect the drug affinity, but enhances the predicted affinity for the natural nucleotide substrates (dATP: −7.40 to −9.10 and dGTP: −9.41 to −12.09). In a simplified model of a single site that binds nucleotides and competing drugs with similar affinities, the molecule with the highest local concentration dictates the outcome. The difference in dosage (10 mg adefovir and 300 mg tenofovir daily) may be important for the evolutionary selection of this resistance mutation. The K212Q substitution affects tenofovir, adefovir, dATP and dGTP affinity values by approximately similar proportions. N236T only affects the value for dGTP binding. Apparently, additional parameters contribute to the viability of K212Q and N236T carrying mutants under an adefovir regime. Thusfar, the S78T and A181I/T/V substitutions might promote tenofovir resistance, but the analysis of the double mutants (S78T+A181T and S78T+A181V) does not indicate a further reduction of tenofovir or adefovir affinities compared with those of dATP and dGTP. For mutants carrying A181T+N236T and A181V+N236T replacements, a slower virological response to tenofovir has been reported [4]. Here, we observed a large reduction in binding affinity of tenofovir and dATP to A181T+N236T mutant RT and a moderate decrease of binding affinity of adefovir and dGTP to A181V+N236T mutant RT. In conclusion, tenofovir and adefovir display different *in silico* interaction profiles with HBV-RT, which points to the presence ((R)-tenofovir) and absence (“rotameric” adefovir) of a center of chirality as documented above. Mutations conferring adefovir resistance in HBV-RT may also affect the tenofovir affinity (particularly S78T and A181T), but in general not to a level that provides tenofovir resistance.

**Table 1:** Protein-ligand energy interaction values of HBV-RT wildtype and mutants associated with adefovir-resistance.

AA replacements	Ref	Mg-Ten	Mg-Ade	Mg-dATP	Mg-dGTP
HBV wt RT		−11.54	−9.10	−7.40	−9.41
N53T	[4]	−10.84	−8.48	−7.57	−8.21
S78T	[4]	−7.48	−8.16	−5.95	−7.01
S85F	[4]	−11.52	−7.54	−6.60	−9.15
A181I	[4]	−7.33	−9.75	−9.55	−7.75
A181T	[10]	−8.28	−5.05	−7.33	−7.11
A181V	[10]	−11.80	−9.23	−9.10	−12.09
K212Q	[4]	−8.27	−6.88	−4.47	−5.85
N236T	[10]	−11.66	−9.38	−7.26	−6.46
A181T+N236T	[4]	−6.18	−9.22	−4.88	−7.29
A181V+N236T	[4]	−11.85	−7.38	−8.12	−8.05
S78T+A181T		−8.41	−7.11	7.65	−7.00
S78T+A181V		−8.33	−10.55	−9.28	−8.83
HIV-1 RT (1HMV chA)		−11.72	−10.17	−8.34	−7.50

The AA replacements were introduced into the wt sequence (type D J02203).

S85F is associated with lamivudine resistance.

Sequences were submitted to i-TASSER for modeling.

PatchDock was used to create protein-ligand complexes.

Energy interaction values (Kcal/mol) were calculated by means of PEARLS.

### 2. Mutational space in view of the overlapping RT and S open reading frames

Open reading frames in the HBV genome show considerable overlap and hence, a drug-resistance mutation in the RT frame might have a negative effect in the overlapping frame encoding the Surface protein [10]. We therefore made an inventory of single amino acid replacements in the HBV-RT sequence associated with adefovir resistance in order to illustrate that detrimental effects in the S-frame are indeed actively avoided (Table 2). For instance, the Asn residue at position 53 is encoded by AAC in wt RT. In an adefovir resistance mutant, a Thr residue (codon ACC) has been selected at this position. When the first nucleotide of the Asn codon (A) is altered into a U, C or G nucleotide, respectively, an Y, H or D residue occurs at this position in HBV RT. In the S-frame, this site corresponds with the 3^rd^ codon position of a residue belonging to the 4-codon family of amino acids and these mutations are therefore synonymous (silent). Mutations at the second codon position will trigger the appearance of I, T or S in the RT enzyme, whereas in the S-protein the original T residue will turn into S, P or A, respectively. In fact, the change in RT from A into T observed *in vivo* is accompanied by an alteration of T into P at the corresponding position in the S-frame. Similar reasoning holds for S85F and K212Q. S78T and A181IVT are much more complex due to involvement of a residue belonging to the 6-codon family of amino acids. Mutation at the first codon position of S78 or A181 into the A-nucleotide in the RT sequence introduces a stopcodon in the S-frame, unless the nucleotide preceding the mutation is a U or C and not a G or A, since both G and A accomplish the introduction of a stopcodon (U**G**A or U**A**A) in the S-frame. Notably, the Asn-to-Thr replacement at position 236 in RT does induce a stopcodon at a position located a few codons downstream of the regular stopcodon of the S reading frame.

**Table 2:** Single nucleotide mutations leading to AA replacements in HBV-RT associated with adefovir resistence.

					Codon-P1				Codon-P2				Codon-P3						
AA	pos	cod	mut		a	u	c	g	a	u	c	g	a	u	c	g			
Asn	53	AAC	T	RevTr		Y	H	D		I	***T***	S	K	N		K			
				Surf	GT	GT	GT	GT	T	S	***P***	A	N	T	T	S			
Ser	78	UCC	T	RevTr	***T***		P	A	Y	F		C	S	S		S	uugACCu	UU(U/C)ACCu	***FT***
				Surf	*P	CP	CP	WP	T	S	C	A	H	L	P	R	uUGACCU	uU(U/C)ACCU	***LP or SP***
Ser	85	UCU	A	RevTr	T		P	***A***	Y	F		C	S		S	S			
				Surf	*L	CL	CL	***WL***	M	L	L	V	Q	L	P	R			
Ala	181	GCU	IVT	RevTr	***T***	S	P		D	***V***		G	A		A	A	cugACUc	CU(U/C)ACUc	***LT***
				Surf	*L	CL	CL	WL	I	***F***	L	V	H	L	P	R	cUGACUC	cU(U/C)ACUC	***LL or SL***
Lys	212	AAG	Q	RevTr		*	***Q***	E		M	T	R	K	N	K				
				Surf	PS	PS	***PS***	PS	S	C	R	G	N	I	S	S			
Val	214	GUA	A	RevTr	I	L	L		E		***A***	G		V	V	V			
				Surf	LY	LY	LY	LY	N	Y	***H***	D	Y	F	S	C			
Gln	215	CAG	S	RevTr	K	*		E		L	P	R	Q	H	H				
				Surf	*S	YS	YS	*S	S	C	R	G	N	I	T	S			
Asn	236	AAC	T	RevTr		Y	H	D		I	***T***	S	K	N		K			
				Surf	*T	YT	YT	*T	*T	*S	*P	*A	*N	*I	*T	*S			

The left 4 columns show the details of the adefovir-associated resistance mutations.

The middle 12 columns (tripartitioned according to codon position) show the effect of each nucleotide substitution in the reverse transcriptase codon on the surface protein.

The change observed in vivo is in bold and italized format.

Sequence details at the right side show how the introduction of a stopcodon in surface protein due to mutation in RT is avoided (capital format is used for codons).

Mutations in codons can be divided into “easy” transitions (A-to-G, C-to-U and vice versa) and “difficult” transversions (nucleotide substitutions between purines and pyrimidines). For example (see Table 2), the N53T substitution involves a “difficult” AAC to ACC transversion like K212Q (AAG to CAG) and N236T (AAC to ACC). S85F (UCU to UUU) represents an “easy” transition that provides lamivudine resistance [4]. The replacements S78T (UCC to ACC) and A181VT (GCU to ACU and GCU to GUU) are also “easy” transitions, but have to avoid the introduction of a stopcodon in the overlapping Surface reading frame. A181I requires “double hit” substitutions at the codon involved. The prevalence of “difficult” transversion type of mutations indicates that “easy” transitions at that codon will not result in a RT enzyme that is both functionally active and drug-resistant [9]. In HBV, the selection of drug-resistant mutations is further constrained by the RT-Surface gene overlap, since functional RT and S proteins are both required for a viable HBV virus (wt or mutant). In HIV-RT, which lacks such a genetic overlap, mutations associated with tenofovir resistance have indeed been reported [11]. Apparently, tenofovir resistance of HBV-RT cannot be attained by “easy” and/or “single-hit” nucleotide substitutions.

### 3. Adefovir, tenofovir and nucleotide binding sites in HBV RT

**Figure 2:**
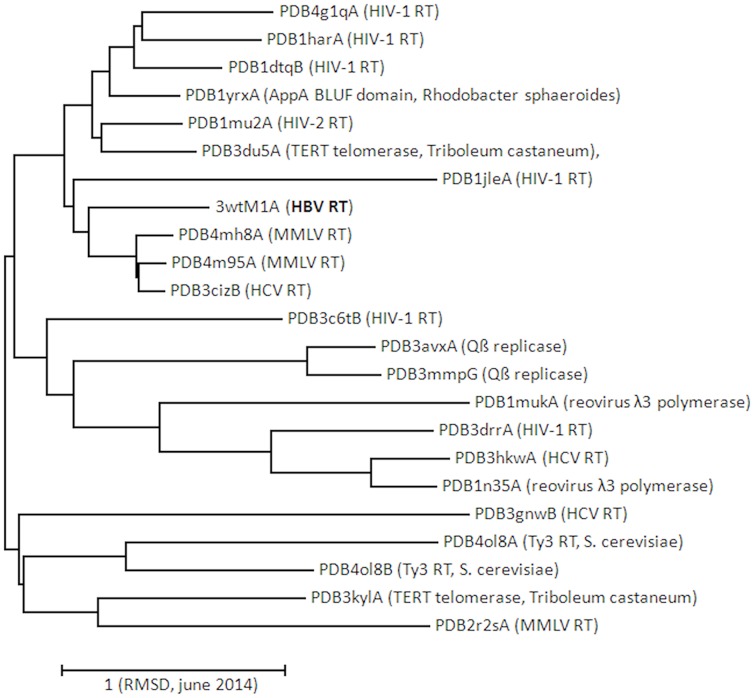
Clustering analysis of *in silico* modeled HBV RT with crystal models of polymerase. The 3D-structure of HBV-RT and the PDB coordinate files of the other polymerases were aligned and the pairwise RMSD values were put into a distance matrix for tree construction. HBV-RT is indicated in bold-face. The scale bar represents a RMSD value of 1. The date is added to anticipate future additions to the PDB database.

A crystal structure of HBV RT is not available, but an *in silico* generated 3D-model has been proposed a few years ago [4]. We applied PDB database screening, 3D-structure alignment and clustering analysis of pairwise RMSD values to investigate similarity of the HBV RT 3D-model with available crystal structures of RT and other polymerases (Figure 2). MMLV and HIV-1 RTs are most prominent among the collection of nearest neighbors, which also includes HIV-2 RT, HCV polymerase, Qß replicase, reovirus λ3 polymerase and three non-viral polymerases. Pairwise RMSD values varied from 0.231 to 4.271 (average 2.868 and standard deviation 0.349). RMSD values of HBV RT paired with the other representatives were between 0.867 and 3.052. On the basis of these results we conclude that the *in silico* generated wt HBV RT model shares considerable similarity with a variety of polymerase structures having coordinate files determined by crystallography, which allowed the identification of amino acid residues or motifs that are involved in the binding of substrates (drugs and nucleotides). The central motif marking this activity is the well-known ^203^YMDD polymerase motif. The PDB coordinate file of wt HBV RT is provided as Data File S1.

**Figure 3:**
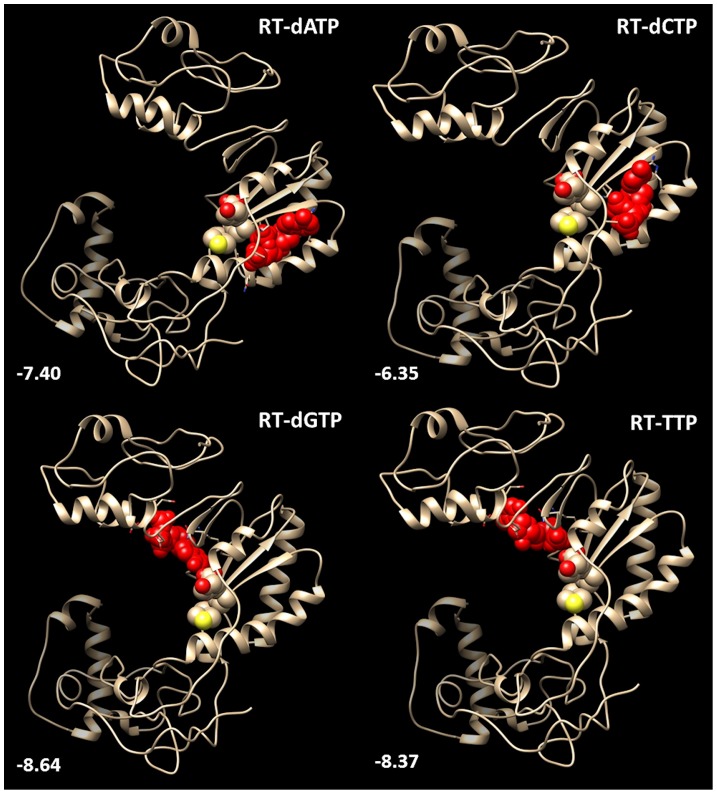
Binding of deoxyribonucleotidetriphosphates to wt HBV reverse transcriptase. The Mg-dNTPs are in red-colored, space-filled format. Other space-filled residues indicate the YMDD motif sequence marking the HBV-RT nucleotide binding site. Numbers in white are the total protein-ligand energy interaction values indicating the Mg-dNTP affinity to wt HBV-RT.

**Figure 4:**
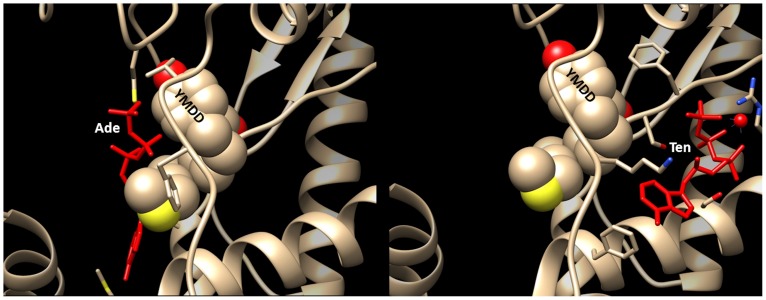
Binding of tenofovir and adefovir to wt HBV reverse transcriptase. Mg-adefovir (left panel, red cartoon) binds close to YMDD (space-filled format) in the large central pocket of HBV-RT. Mg-tenofovir (right panel, red cartoon) binds in a small pocket at the backside of YMDD (space-filled format).

The combination of protein-ligand docking (PatchDock, [12]) and subsequent complex analysis (PEARLS, [8]) allowed the visualization of the most stable complex of wt HBV-RT with each of the four nucleotides (Figure 3). Two distinct candidate binding sites were identified. As expected, dGTP and TTP can bind inside the large central pocket of RT near the Y-residue of the YMDD motif. However, the most stable binding of dATP and dCTP occurs in a small pocket at the backside of the RT structure close to the central residues of YMDD. Predicted binding affinities of −8.64 (dGTP) and −6.35 Kcal/mol (dCTP) are significantly less than the values for tenofovir (−11.54) and adefovir (−9.10) binding to wt RT (Table 1). The difference in binding site does not necessarily reflect the purine/pyrimidine distinction between the nucleotides. The purine analogues adefovir and tenofovir also occupy different positions in close vicinity to the RT YMDD motif (Figure 4). Adefovir is most stably bound at the central pocket of YMDD, like dGTP and TTP. Tenofovir is bound in the small pocket at the backside of the YMDD nucleotide binding motif like dATP and dCTP. Different sites for the most stable binding of tenofovir versus adefovir point to the need for a different mutational profile in the evolution of drug-resistance as documented above (Table 1).

**Figure 5:**
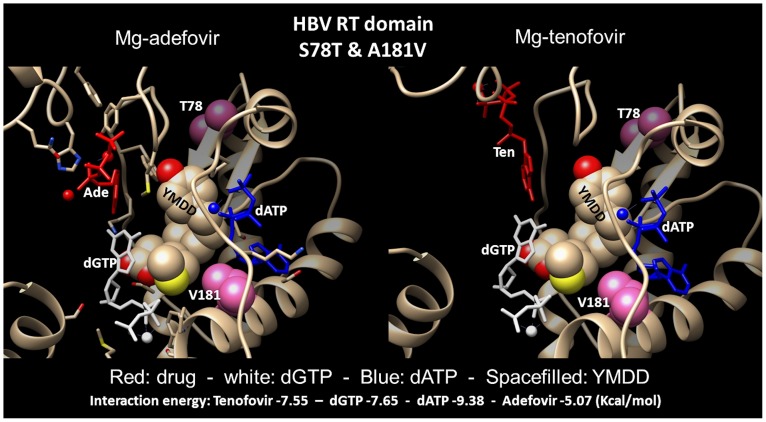
Sequential docking of dATP, dGTP, and adefovir (left panel) or tenofovir (right panel) into the HBV-RT structure carrying S78T & A181V amino acid replacements. T78 and V181 are in hot-pink. YMDD is indicated by space-filling. Mg-adefovir and Mg-tenofovir are in red cartoon format. Mg-dATP is white and Mg-dGTP is blue colored. Total protein-ligand energy interaction values (Kcal/mol) are −9.38 (dATP), −7.65 (dGTP), −7.55 (tenofovir) and −5.07 (adefovir).

Amino acid replacements at HBV-RT positions 78 and 181 (S78T and A181I/T/V, Table 1) seem most promising on the route towards tenofovir resistance. Nucleotide binding sites are often occupied with nucleotides at the onset of drug competition. We therefore challenged the S78T-A181V RT mutant in complex with dATP by docking of dGTP and subsequently either adefovir or tenofovir, and analyzed the resulting complexes for the binding location and stability of the ligands (Figure 5). dATP binding is localized at the same position in mutant and in wt RT, while dGTP has shifted from the N- to the C-terminal side of the YMDD motif sequence. Both adefovir and tenofovir bind to mutant RT near the Y residue of the YMDD motif, a position similar to dGTP binding in wt RT. The S78T and A181V substitutions are positioned slightly remote to the YMDD motif and do not interfere directly with drug or nucleotide binding. The interaction energy profiles show that predicted binding of the ligands, except for dATP, is affected by this way of sequential ligand docking. Most dramatically affected is the binding of adefovir (from −10.55 to −5.07 Kcal/mol), which predicts that the mutant RT resists adefovir action even at high concentrations of the drug.

## Discussion

Tenofovir and adefovir are not administered as triphosphates, but as prodrugs requiring intracellular metabolization. With respect to tenofovir, diesterification of the homochiral 9-[2-(R)-phosphonomethoxypropyl]adenine (PMPA) is a non-stereoselective synthetic route, yielding a 1∶1 mixture of diastereoisomeric pro-drugs due to the asymmetric center at the phosphorus atom. Synthesis and enantiomeric purification of these pro-drugs have been developed into a practical kilo-scale process [13]–[15]. The disoproxil derivative of PMPA (tenofovir) is administered to patients and requires *in vivo* cleavage by diesterases and subsequent phosphorylation by cellular kinases, rendering the biologically active triphosphate compound. It might be expected that nucleotide-using enzymes like DNA-polymerases have a pronounced preference with respect to the spatial conformation of their substrates, although this has not been documented in detail. Adefovir and tenofovir may differ by the rate of bioconversion from pro-drug into bio-active triphosphate. The ß- and γ-phosphates are added by the same enzymatic pathway and the only structural difference between adefovir and tenofovir is the CH_3_-carrying center of asymmetry of tenofovir. We demonstrated a large difference in HBV-RT interaction energy between the (R)- and (S)-isomers of tenofovir, indicating the importance of steric conformation at this center of chirality. Adefovir may be regarded as a collection of rotamers, of which a fraction may energetically be “frozen” into a conformation resembling (S)-tenofovir, thus reducing the affinity for HBV-RT when compared to that of (R)-tenofovir.

Amino acid replacements in HBV-RT cause adefovir resistance and viral breakthrough in patients on antiviral therapy [4], [10]. Tenofovir-related resistance mutations have not yet been reported, even after 6 years of tenofovir monotherapy. Differences in drug dosage applied to patients (10 mg adefovir versus 300 mg tenofovir daily, [16]) may be an obvious reason, particularly in view of the differential interaction with HBV-RT. The higher dosage and binding affinity of tenofovir may be responsible for the more efficient reduction of viral load in patients [16]. The evolutionary selection process required for the induction of resistance may become severely hampered by a significantly larger reduction of the virus' population size under a tenofovir regime compared to adefovir treatment. Tenofovir resistance does not occur either in patients who show a slow decline of HBV viremia after the start of tenofovir therapy [17], [18]. The impact of RT inhibitors in infected cells may become effectively reduced by competition with nucleotide binding sites in unrelated proteins in infected and uninfected cells. For example, adefovir binding to anthrax edema factor effectively inhibits cAMP accumulation in mouse primary macrophages due to the higher affinity of the enzyme for adefovir than for ATP [19].

We used molecular modeling to compare the binding affinities of adefovir and tenofovir to *in silico* generated models of HBV-RT (wt and mutants). The 3D-model of wt HBV-RT displayed significant similarity with a variety of polymerases, indicating a conservation of the overall 3D-structure including the active sites. It should be noted that wt and mutant HBV RT enzymes were independently modeled using the same criteria (constraints and degrees of freedom) instead of homology modeling of mutant RTs on a single wt HBV RT template. Binding affinities of drugs and nucleotides to HBV RTs were estimated by means of the protein-ligand energy interaction profiles [8] and pleiotropic patterns were observed. Single site mutations associated with adefovir resistance affect adefovir affinity and the interaction energy for dGTP (N53T) or dATP (S85T). Also, single site mutated HBV-RT variants that are associated with adefovir resistance showed a decreased affinity for adefovir to a value below that of the nucleotides (A181T) or an increase in the affinity for nucleotides to values near or above that of adefovir (A181V). Most of the mutations known to be associated with adefovir resistance [4], [10] also affect tenofovir affinity, but not to a level near or below the nucleotide affinity values (except S78T and A181I). It should be noted that these values represent a relative measure by which a mutation affects the protein-ligand interaction energy. The combined approach of modeling and interaction energy analysis offers a versatile tool to gain insight into the affinity profiles of substrates and their inhibitors. In this respect, the influence of genotypic amino acid replacements and other natural variations on drug or nucleotide binding may be evaluated by means of this methodology. Additional parameters than the total ligand-receptor interaction energy should be considered to realize a reliable prediction of the resistance phenotype of a specific mutation.

Visualization of the HBV-RT enzyme in complex with its substrates provides a detailed image of nucleotide and drug binding at the YMDD region. The analysis revealed drug and nucleotide binding potency at various locations near the YMDD motif facing the large nucleotide binding pocket. The small binding pocket at the backside of YMDD has preferential affinity for dATP, dCTP and tenofovir. Entecavir is a deoxyguanosine analogue that binds to a similar position in HBV-RT, thus effectively inhibiting DNA chain elongation [20], [21]. The sequential docking of dATP, dGTP and adefovir/tenofovir into the S78T+A181V double mutant puts dATP in the wt-like position, but moves dGTP towards the C-terminal Asp residue of the YMDD motif sequence. Tenofovir and adefovir in the mutant structure occupy a wt-like position like dGTP, with affinities affected by similar proportions compared to those of the wt RT structure. Apparently, many sites in pockets surrounding the YMDD nucleotide binding motif are capable of nucleotide (or drug) binding with different specificity and affinity, causing different mutational profiles for tenofovir versus adefovir. In conclusion, amino acid substitutions may affect the affinity for both tenofovir and adefovir, but in the former case not to an extent needed to facilitate drug-resistance. The difference in binding affinity of tenofovir (more than two orders of magnitude in terms of local concentration), a 30x higher dosage of the (R)-tenofovir enantiomer as compared to ‘rotameric’ adefovir, and the constrained mutational space due to gene overlap in the HBV genome form a tripartite explanation for the absence of resistance against tenofovir despite 6 years of clinical use as monotherapy.

## Materials and Methods

GenBank J02203 provided the genotype D wtHBV sequence. A 3D-model of HBV RT has been published previously [4]. Amino acid replacements used in this study were A181T, A181V, N236T [10] and N53T, S78T, S85F, A181I, K212Q, A181T+N236T, A181V+N236T [4]. In short, wt and mutant HBV RT sequences were submitted to *i*-TASSER [22] for combined homology/ab initio 3D-modeling with HIV-1 RT (PDB id 1JLE) as custom-supplied template structure in addition to PDB structures selected by *i*-TASSER for HBV RT modeling (i.e: 1M8X, RNA binding domain of human Puf protein; 1RW3, MMLV RT; 2ZD1 and 1VRT, both HIV-1 RT). In order to support the validity of the wt HBV RT model, we applied the servers MATRAS [23], DALI [24] and COFACTOR [25] screening the (non-redundant) PDB database for crystal structures with significant identity with the *in silico* generated 3D-model of HBV RT. The top10 hits were combined and after removal of duplicates aligned according to 3D-structure similarity (PDBeFOLD, http://www.ebi.ac.uk/msd-srv/ssm/). The pairwise distance matrix of RMSD values was fed into MEGAv6 for nearest-neighbor clustering analysis [26]. The PDB coordinate file of wtHBV RT is provided as Data File S1. The structures of tenofovir, adefovir, dATP, dGTP, dCTP and TTP were taken from the PDB IDs 2FKB, 1ZOT, 3KK2, 1XJJ, 3GQC and 2QXX, respectively. CORINAv3.4 (http://www.molecular-networks.com/online_demos/corina_demo) was used to generate the structure of (S)-tenofovir. PatchDock (based on shape complementarity) [12] was used for *in silico* docking of ligands (protein-small ligand docking, 1.5 Å clustering RMSD) into the RT structure providing the YMDD motif as a mark of the protein's nucleotide binding site. The top 20 of the docking results were submitted to PEARLS [8] for the determination of protein-ligand energy interaction profiles in order to select the complex with the lowest value for total energy interaction predicting the highest binding affinity. Display of structures was done by means of UCSF Chimera V1.8 [27].

## Supporting Information

Data File S1 **Structure of HBV RT.** The structure of reverse transcriptase of hepatitis B virus is provided as a coordinate file in PDB format.
(PDB)
